# Biocontrol Ability and Mechanism of a Broad-Spectrum Antifungal Strain *Bacillus safensis* sp. QN1NO-4 Against Strawberry Anthracnose Caused by *Colletotrichum fragariae*

**DOI:** 10.3389/fmicb.2021.735732

**Published:** 2021-09-17

**Authors:** Xiaojuan Li, Miaoyi Zhang, Dengfeng Qi, Dengbo Zhou, Chunlin Qi, Chunyu Li, Siwen Liu, Dandan Xiang, Lu Zhang, Jianghui Xie, Wei Wang

**Affiliations:** ^1^Key Laboratory of Biology and Genetic Resources of Tropical Crops, Ministry of Agriculture, Institute of Tropical Bioscience and Biotechnology, Chinese Academy of Tropical Agricultural Sciences, Haikou, China; ^2^Ministry of Education Key Laboratory for Ecology of Tropical Islands, College of Life Science, Hainan Normal University, Haikou, China; ^3^College of Ecology and Environment, Hainan University, Haikou, China; ^4^Key Laboratory of South Subtropical Fruit Biology and Genetic Resource Utilization, Ministry of Agriculture, Key Laboratory of Tropical and Subtropical Fruit Tree Research of Guangdong Province, Institution of Fruit Tree Research, Guangdong Academy of Agricultural Sciences, Guangzhou, China

**Keywords:** *Bacillus safensis*, *Colletotrichum fragariae*, biological control, antifungal properties, strawberry fruit, whole genome sequencing

## Abstract

Strawberry is a very popular fruit with a special taste, color, and nutritional value. Anthracnose caused by *Colletotrichum fragariae* severely limits fruit shelf life during post-harvest storage. Use of traditional chemical fungicides leads to serious environment pollution and threatens food safety. Biocontrol is considered as a promising strategy to manage the post-harvest fruit diseases. Here, strain QN1NO-4 isolated from noni (*Morinda citrifolia* L.) fruit exhibited a high antifungal activity against *C. fragariae*. Based on its physicochemical profiles and phylogenetic tree of the 16S *rRNA* sequence, strain QN1NO-4 belonged to the genus *Bacillus*. The average nucleotide identity (ANI) calculated by comparing two standard strain genomes was below 95–96%, suggesting that the strain might be a novel species of the genus *Bacillus* and named as *Bacillus safensis* sp. QN1NO-4. Its extract effectively reduced the incidence of strawberry anthracnose of harvested fruit. Fruit weight and TSS contents were also maintained significantly. The antifungal mechanism assays indicated that the extract of the test antagonist inhibited mycelial growth and spore germination of *C. fragariae in vitro.* Cells of strain QN1NO-4 demonstrated the cytoplasmic heterogeneity, disappeared organelles, and ruptured ultrastructure. Notably, the strain extract also had a broad-spectrum antifungal activity. Compared with the whole genome of strain QN1NO-4, several functional gene clusters involved in the biosynthesis of active secondary metabolites were observed. Fifteen compounds were identified by gas chromatography–mass spectrometry (GC-MS). Hence, the fruit endophyte *B. safensis* sp. QN1NO-4 is a potential bio-agent identified for the management of post-harvest disease of strawberry fruit.

## Introduction

Strawberry (*Fragaria* × *ananassa* Duch.) is one of the most popular fruits with more than 6.1 million tons of annual production in the world (Han et al., 2016; Zhimo et al., 2021). Strawberry fruit is also highly perishable due to mechanical injury and pathogen infection, which limits its shelf life during storage (Rico et al., 2019). Strawberry anthracnose is one of the most serious fungal diseases. It is caused by different *Colletotrichum* fungal species including *Colletotrichum fragariae*, *C. acutatum*, and *C. gloeosporioides* (Denoyes-Rothan et al., 2003; Han et al., 2016). Among the causal agents, *C. fragariae* is an important fungus which causes the disease named anthracnose crown rot (Miller-Butler et al., 2018). The fungi can infect the whole strawberry plant containing fruit, and it has become more destructive in the past decade (Chen et al., 2019; Chung et al., 2020). Currently, the control of strawberry anthracnose is done primarily through chemical fungicides to reduce post-harvest losses (Dukare et al., 2019). However, excessive use of fungicides causes environment pollution and pathogenic resistance (Li et al., 2021; Wang et al., 2021). In some developed countries, use of fungicides has become increasingly limited and even banned (Wisniewski et al., 2016; Wang et al., 2018). Therefore, it is necessary to develop a safer and eco-friendly strategy to manage post-harvest diseases of fruit.

Application of biocontrol agents was considered as an alternative and promising strategy to control plant pathogens (Feliziani et al., 2016; Ye et al., 2021). Biocontrol contributed to minimize the use of chemical pesticides and reduce environmental pollution. In the past few decades, some microorganisms such as yeasts, *Pseudomonas fluorescens*, *Streptomyces* spp., and *Bacillus* species were accepted as important biocontrol agents (Rong et al., 2020; Einloft et al., 2021). They had potential as an alternative to synthetic fungicides. The well-studied *Pichia membranefaciens* and *Pichia guilliermondii* were effective in controlling rhizopus rot of peaches and anthracnose of loquat fruit (Zhao et al., 2019; Zhang et al., 2020). Recent studies demonstrated that *Pseudomonas synxantha* had a biocontrol efficacy against *Monilinia fructicola* and *Monilinia fructigena* in stone fruit (Aiello et al., 2019). *P. fluorescens* can suppress post-harvest gray mold in apples (Zhang et al., 2011; Wallace et al., 2018).

Especially, the Bacillus genera were evaluated as outstanding biocontrol agents against fungal pathogens, such as *Bacillus halotolerans* against *Botrytis cinerea* in strawberry fruit (Wang et al., 2021; Ye et al., 2021), *Bacillus* sp. w176 against post-harvest green mold in citrus (Tian et al., 2020), *Bacillus atrophaeus* against anthracnose in soursop and avocado (Guardado-Valdivia et al., 2018), and *Bacillus amyloliquefaciens* against other phytopathogens of fruits (Calvo et al., 2017; Gotor-Vila et al., 2017; Wang et al., 2020; Ye et al., 2021). Several *Bacillus* spp. had been applied as biofertilizers or biopesticides in different crops (Li et al., 2019). They inhibited the pathogenic growth and improved the plant resistance by competition for space or nutrients with pathogens and production of bioactive substances and cell wall-degrading compounds (Ye et al., 2021). Although various *Bacillus* sp. strains are reported in biocontrol of post-harvest fruit, little work is conducted to identify a broad-spectrum antifungal *Bacillus* strain with an increased efficiency.

In our study, a strain marked with QN1NO-4 with a wide-spectrum antifungal ability is newly isolated from noni fruit. Based on the physicochemical characteristics as well as average nucleotide identity (ANI) assay, the strain is identified as a species of the genus *Bacillus*, called after *Bacillus safensis* sp. QN1NO-4. The isolated extracts successfully inhibit the infection of *C. fragariae* on strawberry fruit during post-harvest and could effectively keep the weight loss and TSS of fruit. The antifungal mechanism is investigated by assaying its effects on spore germination and morphological profile by scanning electron microscopy (SEM) as well as hyphal ultrastructure of *C. fragariae* by transmission electron microscopy (TEM). The complete genome of strain QN1NO-4 reveals a number of key gene clusters of active secondary metabolites. Antimicrobial compounds of strain QN1NO-4 extracts are further identified by gas chromatography–mass spectrometry (GC-MS). These results indicate that the fruit endophyte *B. safensis* sp. QN1NO-4 is a promising biocontrol agent for controlling post-harvest diseases of strawberry fruit.

## Materials and Methods

### Fruit Materials

Strawberry (*Fragaria* × *ananassa* Duch var. Zhang Ji) fruit with similar size (approximately 5-cm diameter), color, and shape was selected from a supermarket. No visible injury and pathogen infection were detected on the fruit surface. After being surface-sterilized with 75% (v/v) of ethanol for 2 min, these fruit samples were rinsed with sterile water for three times and air-dried for 2 h at 25°C.

Noni (*Morinda citrifolia* L.) fruit was collected from a planting farm in Chengmai city (19°58′35″N, 109°55′35″E) of Hainan province, China. Fruit samples were surface-sterilized with 75% (v/v) of ethanol for 5 min, disinfected using 2% of sodium hypochlorite for 20 min, and washed using sterile water for five times.

### Bacterial Isolation

Ten grams of noni fruit was ground in a sterile mortar until paste. One milliliter of homogenate was mixed with 4 ml of Luria–Bertani (LB) medium into a 50-ml conical flask. The mixture was cultured with shaking at 180 rpm for 1 h at room temperature. The suspension was diluted from 10^–1^ to 10^–3^ fold with sterile distilled water. Two hundred microliters of dilution was spread on the LB solid medium and incubated at 28°C for 2 days. A single colony was obtained by repeatedly streaking on LB plates and was kept in 20% (v/v) glycerol at −80°C.

### Screening of Biocontrol Bacteria Against *C. fragariae*

Fungal pathogen *C. fragariae* (ATCC 58718) was kindly provided by the Institute of Environment and Plant Protection, China Academy of Tropical Agricultural Sciences, Haikou, China. The antifungal ability of each isolated bacterium was tested against *C. fragariae* using our reported method (Li et al., 2021). Briefly, fungal pathogen was first cultured on a potato dextrose agar (PDA) plate for 7 days. A 5-mm-diameter pathogenic disk was prepared and placed in the middle of the plate. Each isolated bacterium was spotted on the four symmetrical points of the pathogen. The fungal disk alone was used as a control. Five Petri dishes were used for each replicate with a diameter of 90 mm. After culture at 28°C for 7 days, antifungal activity of the isolated bacterium was assessed by determining the radial mycelial growth of fungal pathogen. To further detect the antifungal activity of the isolated bacterium supernatant, the isolate was inoculated in 50 ml of LB liquid medium in a 250-ml conical flask. After being cultured at 150 rpm and 28°C for 3 days, the supernatant was filtered using the Whatman no. 1 qualitative filter paper and sterilized through a 0.22-μm sterile filter (Millipore, Bedford, MA, United States). Twenty microliters of supernatant was spotted at four symmetrical points of the tested pathogens, respectively. An equal amount of LB was used as a control. The antagonistic experiment was kept at 28°C for 7 days. The antifungal activity of the isolated bacterium supernatant was assessed by determining the radial mycelial growth of the fungal pathogen. All experiments were carried out in triplicate.

### Morphological, Physiological, and Biochemical Characteristics of the Selected Isolate

Morphological profiles of the selected isolate were observed after growth at 37°C for 3–4 days. Physiological and biochemical indexes were tested including resistance to pH, temperature and NaCl, enzymatic characteristics, and utilization of carbon and nitrogen sources (Wei et al., 2020). In addition, a disk diffusion method was applied to test the sensitivity of the selected isolate to different antibiotics (Kumar et al., 2014).

### Antifungal Activity Assays of Extract From the Selected Isolate

Extract was prepared according to the previous description with a minor modification (Qi et al., 2019). Briefly, the selected isolate was inoculated in a 5-l Erlenmeyer flask containing 1 l of LB liquid medium. After culture in a rotary shaker (180 rpm) for 3 days at 28°C, an equal volume of ethyl acetate was added to the supernatant. The mixture was subjected to ultrasound for 1 h and then injected into a separating funnel. The organic solvent in the collected extract was evaporated using a rotary vacuum evaporator (N-1300, EYELA, Ailang Instrument Co., Ltd., Shanghai, China). The dried extract was then dissolved in 100% of methanol, and 10 g l^–1^ of stock solution was prepared. To detect the antifungal activity assays of the extract, a 5-mm-diameter pathogenic disk was placed in the middle of the PDA plate containing 50 mg l^–1^ of extract. After culture at 28°C for 7 days, antifungal activity was assessed by determining the radial mycelial growth of fungal pathogen. All experiments were carried out in triplicate.

### Inhibitory Efficiency of Extract on Mycelial Growth of *C. fragariae*

The stock solution (10 g l^–1^) was diluted into 5, 2.5, 1.25, 0.625, 0.313, 0.156, and 0.078 g l^–1^ using a double continuous dilution method (Wei et al., 2020). When 50 ml of the sterilized PDA liquid medium was precooled to 40–50°C, 1 ml of each extract diluent was added to generate different treatment concentrations (1.563, 3.125, 6.25, 12.5, 25, 50, 100, and 200 mg l^–1^, respectively). The inhibition ability of each extract concentration on the mycelial growth of *C. fragariae* was analyzed according to our previous method (Li X.J. et al., 2020). Antifungal activity was measured according to the growth diameter of *C. fragariae* at 28°C for 7 days. The morphological profile of pathogenic mycelia treated with 200 mg l^–1^ of extract was observed using an optical microscope (Nikon, E200MV, Japan). The half-maximal effective concentration (*EC*_50_) of the extract against *C. fragariae* was calculated in the light of a toxicity regression equation established by a least square method (Vanewijk and Hoekstra, 1993).

### Inhibitory Efficiency of Extract on Controlling Decay of Strawberry Fruit

Different fold *EC*_50_ concentrations (2 × *EC*_50_, 4 × *EC*_50_, and 8 × *EC*_50_) were first prepared. Based on the extract quantity of the 1 × *EC*_50_ value, the proper volume of the stock solution (10 mg ml^–1^) was diluted into 8 × *EC*_50_ with sterile distilled water. Then, the double continuous dilution method was used to obtain 4 × *EC*_50_, 2 × *EC*_50_, and 1 × *EC*_50_, respectively. The antagonistic ability of the extract was tested for controlling post-harvest anthracnose decay of strawberry fruit according to our previous description (Li et al., 2021). A 2-mm-wide and 1-mm-deep wound was manufactured on the surface of the selected fruit samples using a sterilized needle. Ten microliters of different concentrations of extracts (1 × *EC*_50_, 2 × *EC*_50_, 4 × *EC*_50_, and 8 × *EC*_50_, respectively) was inoculated into fruit wound. An equal volume of sterile water was used as a control. After the treated fruits were air-dried, 10 μl of conidial suspension (1.0 × 10^6^ CFU ml^–1^) was added to the wounded site. All fruit samples were kept in an artificial climate cabinet (Ever Scientific Instrument Ltd., Shanghai, China) at 28°C and 85% relative humidity under 12 h light/12 h darkness for 7 days. Twenty-four fruit samples of strawberry were selected for each treatment in three replicates. The disease incidence (DI) was calculated according to the following formula:


(1) DI(%)=(D1/D2)×100

where D1 and D2 represent the number of decayed fruits and total fruits, respectively.

### Preservative Effects of Extract on Quality Parameters of Strawberry Fruit

The weight loss and total soluble solids (TSS) of strawberry fruit were determined after treatment with *C. fragariae* and different concentration extracts of the isolated strain for 7 days (Li et al., 2021). Weight loss was expressed as a percentage of fruit weight before and after storage. TSS content was measured using the refractive index of fruit juice with a MASTER Refractometer (PAL-1, Atago, Japan).

### Antifungal Activity of Secondary Metabolites Against *C. fragariae*

The fungal spores were collected from the PDA medium by rubbing and washing the surface of each petri dish with a sterile L-shaped spreader. The mycelia were removed using a sterile muslin cloth. The spore concentration was adjusted to 1.0 × 10^6^ CFU ml^–1^ with a hemocytometer (Neubauer, Superior Ltd., Marienfeld, Germany). An equal volume of pathogenic spore suspension (1.0 × 10^6^ CFU ml^–1^) and each concentration extract (1 × *EC*_50_, 2 × *EC*_50_, 4 × *EC*_50_, or 8 × *EC*_50_, respectively) was mixed at 28°C and 85% relative humidity for 12 h. More than 200 spores of *C. fragariae* were used to detect germination using an optical microscope (Nikon, E200MV, Japan) (Pei et al., 2020). Based on the spore germination, 4 × *EC*_50_ of extract concentration was selected for the following experiment.

### Morphological Profile of *C. fragariae* Spores Treated With Extract

To detect the effect of extract on the spore morphology of *C. fragariae*, equal volumes of spores (1.0 × 10^6^ CFU ml^–1^) and 4 × *EC*_50_ of extracts were completely mixed in a 10-ml sterile centrifugal tube. The mixture was incubated at 28°C for 6 h. After centrifugation at 10,000 rpm for 5 min at 4°C, spores were collected and fixed with 2.5% (v/v) of glutaraldehyde overnight. Then, these fixed spores were washed for three times with a phosphate-buffered saline (PBS, 0.1 mol l^–1^, pH 7.2) for 15 min. The sample was dehydrated using a gradient ethanol solution (30, 50, 70, 80, 90, 95, and 100%, 15 min for each time). The dried samples were coated with gold powder using the ion sputtering instrument (Qi et al., 2019). Platinum was used as the plating material. Samples coated with a film of gold–palladium alloy under vacuum were detected by a scanning electron microscope (SEM, Sigma 500/VP, Zeiss, Germany).

### Ultrastructural Profile of *C. fragariae* Cells Treated With Extract

*C. fragariae* was cultured in the sterilized PDA liquid medium at 28°C and 200 rpm for 5 days. Cells of fungal pathogen were collected and fixed as described in Section “Morphological Profile of *C. fragariae* Spores Treated With Extract.” The pathogenic samples were embedded in Epon 812 resin. An 80-nm-thick section was manufactured by an ultramicrotome (EM UC6, Leica, Germany). The sections were stained with 3% (v/v) of lead citrate for 10 min and 3% (v/v) of saturated uranyl acetate for 30 min. The cell ultrastructure of *C. fragariae* was observed by transmission electron microscopy (TEM, HT7700, Hitachi, Japan).

### Hemolytic Assay of Human Red Blood Cells Treated With Extract

To test the safety of the extract for human, a hemolysis experiment was performed (Li et al., 2021). The human red blood cells were collected by centrifugation at 1,000 *g* for 5 min and then were washed with 0.85% (w/v) of normal saline for three times. Two percent of red blood cells was prepared as in our previous description (Li et al., 2021). Four hundred fifty microliters of blood cell solution was mixed with 50 μl of extract (1 × *EC*_50_, 2 × *EC*_50_, 4 × *EC*_50_, or 8 × *EC*_50_) at 37°C for 1 h. The red blood cells treated with 0.85% (w/v) of normal saline and 0.1% (v/v) of Triton X-100 were used as negative and positive controls, respectively. The release of hemoglobin was monitored by absorbance at 540 nm. Hemolytic activity was expressed as the percentage of total hemoglobin released by extract treatment in comparison with that released by Triton X-100 treatment (Wang et al., 2018).

### Assay of a Broad-Spectrum Antifungal Activity of the Selected Isolate Extract

In order to test whether the selected isolate extract had a broad-spectrum antifungal activity, we selected 10 phytopathogenic fungi, including *C. fragariae* (ATCC 58718), *Fusarium graminearum* (ATCC MYA-4620), *C. acutatum* (ATCC 56815), *F. oxysporum* f. sp. *cucumerinum* (ATCC 204378), *Curvularia lunata* (ATCC 42011), *Pyricularia oryzae* (ATCC 52352), *C. gloeosporioides* Penz. (ATCC 58222), *Alternaria* sp. (ATCC 20492), *C. capsici* (ATCC 48574), and *C. gloeosporioides* (ATCC 16330). These phytopathogenic fungi were kept in our lab. The antifungal activity of the extract was detected against each phytopathogenic fungus according to our previous method (Wei et al., 2020).

### Genome Sequencing and Metabolite Prediction of the Selected Isolate

The genomic DNA of the selected isolate was extracted using the BioTeke Bacterial Genomic DNA Rapid Extraction Kit (DP1301, Beijing Biotech Co., Ltd., China) and quantified using the TBS-380 fluorometer (Turner BioSystems Inc., Sunnyvale, CA, United States). Sequencing was performed on the platform of the Illumina high-throughput sequencing platform (HiSeq). The complete genome was assembled by Majorbio Bio-Pharm Technology Co., Ltd., Shanghai, China. Bioinformatics analysis was carried out using the I-Sanger Cloud Platform 2.0. The ANI was analyzed using the online OrthoANI (Yoon et al., 2017). The G + C content of the strain genome was calculated based on the complete genome sequence. The protein-coding genes were predicted by the Glimmer v3.02 software (Delcher et al., 2007). The unigenes were annotated using the public databases of Gene Ontology (GO^1^), Clusters of Orthologous Groups of proteins (COG^2^), and Kyoto Encyclopedia of Genes and Genomes (KEGG^3^) (Zhang et al., 2020). Gene cluster analysis of secondary metabolite synthesis was carried out by the online antiSMASH v4.2.0 software (Huang et al., 2019).

### Identification of Chemical Compounds From Extracts

The chemical compounds of the extract were identified by gas chromatography–mass spectrometry (GC-MS) (Ogundajo et al., 2017). The analysis was performed on gas chromatography (5973 Inert XL MSD, Agilent, United States) plus with DB-FFAP (30 m × 0.25 mm × 0.25 μm) and a triple quadrupole mass spectrometer. Helium was used as a carrier gas at 1 ml min^–1^. The temperature program was kept at 70°C for 2 min and increased to 100°C at 5°C min^–1^ for 5 min, 250°C at 10°C min^–1^ for 35 min, and 280°C at 4°C min^–1^ for 5 min. The mass spectrometer was operated in an optimized condition as in our previous description (Li et al., 2021); these data of mass spectra were determined by alignment with the National Institute of the Standards and Technology (NIST) spectral library version 2.0.

### Data Analysis

A one-way analysis of variance (ANOVA) was applied to analyze all data using the SPSS software version 22 (SPSS Inc., Chicago, IL, United States). Data from three independent experiments were expressed as the mean ± standard deviation. A significant difference was evaluated in the light of the LSD multiple-range test at the level of *p* < 0.05.

## Results

### Isolation and Screening of Biocontrol Bacteria

**FIGURE 1:**
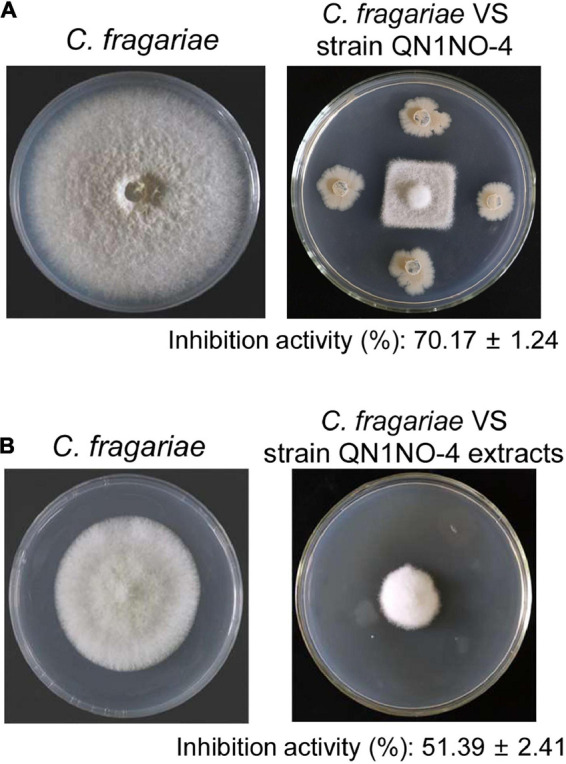
Antifungal activity of strain QN1NO-4 and its extract against *C. fragariae* of strawberry. **(A)** *In vitro* inhibition activity of strain QN1NO-4 on mycelial growth of *C. fragariae*. **(B)** Effects of extract isolated with 100% of methanol on mycelial growth of *C. fragariae*.

A total of 35 endophytic bacteria were isolated from noni fruit. Twenty-nine bacteria had an antifungal activity against *C. fragariae in vitro* in Supplementary Table 1. The fermentation broth of 10 endophytic bacteria exhibited antagonistic activity against *C. fragariae* (Supplementary Table 2). Especially, a strain labeled with QN1NO-4 had the strongest antagonistic activity. Compared with 85.50 mm ± 1.43 of growth diameter of *C. fragariae* in the control plate, the growth diameter was 25.50 mm ± 0.79 in the presence of strain QN1NO-4 (Figure 1A). The inhibition activity of mycelial growth was up to 70%. To further analyze antifungal components of strain QN1NO-4, extracts were isolated using 100% of methanol. The inhibition activity was approximately 51% in the PDA solid medium with 50 mg l^–1^ of the final extract concentration (Figure 1B). Hence, strain QN1NO-4 was selected for the following study.

### Identification and Genome Sequencing of Strain QN1NO-4

**FIGURE 2:**
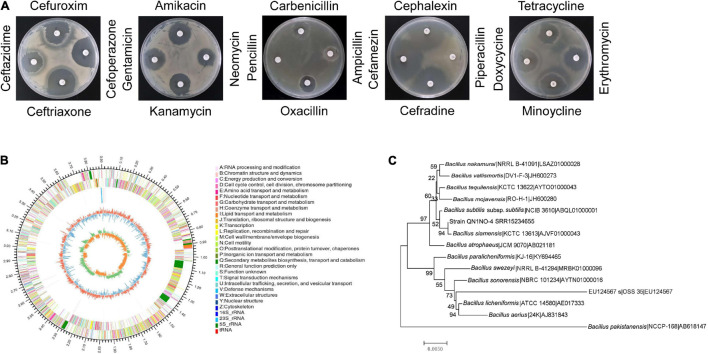
Identification of strain QN1NO-4. **(A)** Antibiotic sensitivity test of strain QN1NO-4. **(B)** Circular map of the strain QN1NO-4 genome. From outside to center, ring 1 represented the genome-size marker. Rings 2 and 3 showed coding sequences on forward and reverse strands, respectively. Ring 4 demonstrated rRNAs and tRNAs. Ring 5 represented the G + C content, followed by G + C skew in ring 6. **(C)** Phylogenetic tree of strain QN1NO-4 based on the 16S *rRNA* sequence. The tree was constructed using the neighbor-joining method in the MEGA software. The level of bootstrap support (1,000 repetitions) was indicated at all nodes.

Based on the analysis of the morphological properties, strain QN1NO-4 was a round and primrose yellow colony on the LB plate. The colonial margin became much rougher and more irregular along with aging. The physiological and biochemical characteristics showed that strain QN1NO-4 could produce urease, catalase, and nitrate reductase but could not generate hydrogen sulfide. Positive results were detected under analysis of gelatin liquefaction, starch hydrolysis, or *V-P* test. The strain could grow on the medium with up to 13% of NaCl, temperature from 30 to 65°C, and pH from 5 to 10. It could utilize all carbon sources and nitrogen sources tested except for L-glutamate and L-tyrosine. In addition, strain QN1NO-4 was sensitive to 18 antibiotics tested except for penicillin and piperacillin (Table 1; Figure 2A and Supplementary Tables 3, 4).

**TABLE 1:** Physiological and biochemical characteristics of strain QN1NO-4.

Characteristics	Result
*Biochemical test*	
Urease production	+
Tween-20	–
Tween-40	–
Tween-60	–
Catalase	+
Oxidase	–
Gelatin liquefaction	+
Starch hydrolysis	+
Decomposition of cellulose	–
Nitrate reduction	+
H_2_S production	–
*MR* test	–
*V-P* test	+
*Physiological test*	
pH tolerance	5–10 (optimum 7)
NaCl tolerance (%)	1–13 (optimum 5)
Temperature tolerance (°C)	30–65 (optimum 37)

*+, positive reaction; –, negative reaction.*

The complete genome of strain QN1NO-4 was sequenced and submitted to the GenBank with accession number of SRR15234655. It contained 3,923,715 bp with 46% of G + C content. The genome consisted of three rRNA genes, 52 tRNA genes, and 3,917 protein-coding genes (Figure 2B and Table 2). Based on 16S *rRNA* gene sequences, a phylogenetic tree was constructed using the neighbor-joining method (Figure 2C). Strain QN1NO-4 showed a 100% of similarity with the standard strain *B. siamensis* (KCTC 13613). In addition, the ANI value was calculated to further identify the species of strain QN1NO-4. Genomic data of the highest homology standard strains (*B. siamensis* KCTC 13613 and *B. subtilis* NCIB 3610) were downloaded from the EzBioCloud public genome database^4^ and then were submitted to the ANI computing platform.^5^ The ANI values of strain QN1NO-4 were 94 and 77% in comparison with two standard strains *B. siamensis* KCTC 13613 and *B. subtilis* NCIB 3610, respectively (Supplementary Table 5). Both ANI values were below 95–96% of the threshold value for novel species definition (Richter and Rosselló-Móra, 2009). Thus, strain QN1NO-4 might be a novel species of the genus *Bacillus*, called after *B. safensis* sp. QN1NO-4.

**TABLE 2:** Summary of the strain QN1NO-4 genome.

Attribute	Value	% of total
Genome size (bp)	3,923,715	100
DNA coding region (bp)	3,428,622	87.38
DNA G + C content (bp)	1,824,135	46.49
Total genes	3,972	100
tRNA genes	52	1.31
rRNA genes	3	0.08
Protein-coding genes	3,917	98.62
Genes assigned to COGs	2,967	75.75
Genes assigned to GO	2,554	65.20
Genes assigned to KEGG	2,190	55.91
CRISPR repeat	10	0.26

### Determination of the Half-Maximal Effective Concentration Value of the Extract Against *C. fragariae*

**FIGURE 3:**
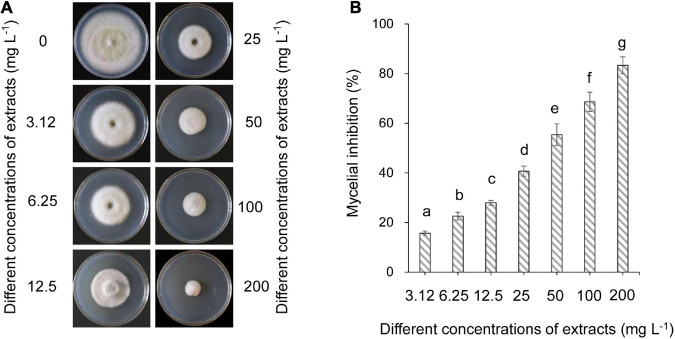
Determination of the half-maximal effective concentration value of extract against *C. fragariae*. **(A)** Growth inhibition of *C. fragariae* on the PDA medium after treatment with different dose extracts. **(B)** Quantitative analysis of *C. fragariae* growth diameters. Error bars indicated standard errors of the means of three repeated experiments. Different lowercase letters represented a significant difference (*p* < 0.05) of mycelial growth diameter at the same time point using the LSD multiple-range test.

The effects of strain QN1NO-4 extracts on mycelial growth of *C. fragariae* were assayed. The inhibitory efficiency showed a dose-dependent manner (Figure 3A). After the strain QN1NO-4 extract was co-incubated with *C. fragariae* at 28°C for 7 days, significant growth inhibition of the pathogen was observed in all the concentration groups (1.563, 3.125, 6.25, 12.5, 25, 50, 100, and 200 mg l^–1^). The inhibitory efficiency was 16, 23, 28, 41, 55, 69, and 83%, respectively (Figure 3B). Compared with the growth diameter of *C. fragariae* (68.00 mm ± 0.1) in the control plate, the half-maximal effective concentration (*EC*_50_) value of the strain QN1NO-4 extract was 33.81 ± 0.46 mg l^–1^ using the toxicity regression equation. The concentration was defined as 1 × *EC*_50_ for the following study.

### Biocontrol Efficiency of Extract on Controlling Strawberry Anthracnose Caused by *C. fragariae*

**FIGURE 4:**
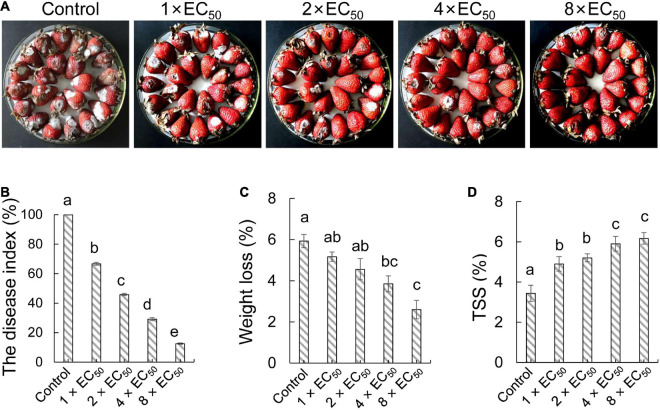
*In vivo* effects of strain QN1NO-4 extract on fruit decays and disease incidence caused by *C. fragariae*. **(A)** Disease inhibition of extract on strawberry fruit. Four extract concentrations (1 × *EC*_50_, 2 × *EC*_50_, 4 × *EC*_50_, and 8 × *EC*_50_) were selected. The treated fruit samples were stored at 28°C for 7 days. **(B)** Quantitative analysis of disease incidence of strawberry fruit treated with different dose extracts. **(C)** Effects of strain QN1NO-4 extract on weight loss of strawberry fruit. **(D)** Effects of strain QN1NO-4 extract on TSS contents of strawberry fruit. Error bars indicated standard errors of the means of three repeated experiments. Different letters indicated a significant difference (*p* < 0.05) among different dose extracts using the LSD multiple-range test.

To investigate the biocontrol efficiency of strain QN1NO-4 against *C. fragariae* in post-harvest strawberry, each concentration extract (1 × *EC*_50_, 2 × *EC*_50_, 4 × *EC*_50_, or 8 × *EC*_50_, respectively) was used to treat the selected fruit samples (Figure 4A). After artificially co-incubating with *C. fragariae* (1.0 × 10^6^ CFU ml^–1^) for 7 days, the DIs and quality parameters of strawberry fruit were measured. Compared with the control group, all treatments alleviated significantly the infection of *C. fragariae* in post-harvest strawberry fruit at 7 days post-inoculation (dpi). The DIs were 67, 46, 29, and 13% in treatment groups of 1 × *EC*_50_, 2 × *EC*_50_, 4 × *EC*_50_, and 8 × *EC*_50_, respectively (Figure 4B). Therefore, strain QN1NO-4 extract can inhibit effectively the infection of *C. fragariae* in post-harvest strawberry fruit.

Weight loss and TSS were measured to evaluate the effects of the strain QN1NO-4 extract on the fruit quality of strawberry. Weight loss showed a continuous decrease along with the increasing dose of extract (Figure 4C). Compared with the control group, weight loss was reduced by 4 and 3% after treatment with 4 × *EC*_50_ and 8 × *EC*_50_, respectively. No obvious difference was observed between 1 × *EC*_50_ and 2 × *EC*_50_ treatment groups. Moreover, strain QN1NO-4 extract also delayed the decrease in TSS content during storage. Compared with the control group at 7 dpi, high TSS contents were kept in the treated fruit samples (Figure 4D).

### Effects of Strain QN1NO-4 Extract on Spore Germination, Morphological Profile, and Cell Ultrastructure of *C. fragariae*

**FIGURE 5:**
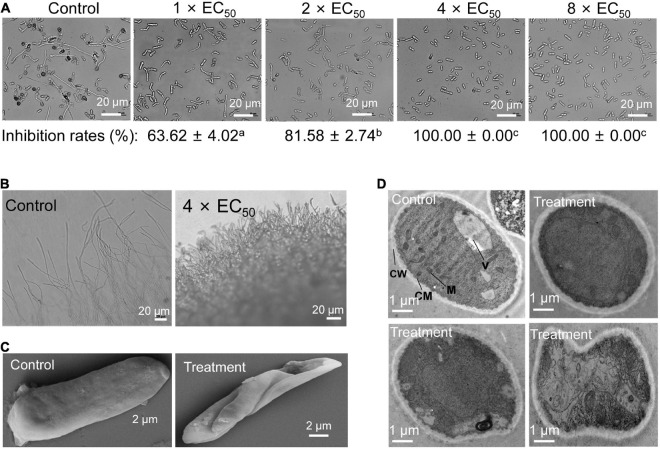
Inhibitory effects of strain QN1NO-4 extract on conidial germination, mycelial morphology, and ultrastructure of *C. fragariae.* **(A)** Conidial germination assay of *C. fragariae* after treatment with 1×, 2×, 4×, or 8 × *EC*_50_ extracts. Bar = 20 μm. CW: cell wall; CM: cell membrane; M: mitochondria; V: vacuole. **(B)** Growth characteristics of *C. fragariae* mycelia treated with 4 × *EC*_50_ of extract. Bar = 20 μm. **(C)** Representative pictures showing the mycelial morphology of *C. fragariae* after treatment with 4 × *EC*_50_ of extracts. Bar = 20 μm. **(D)** Representative pictures showing the ultrastructure of *C. fragariae* after treatment with 4 × *EC*_50_ of extract. Bar = 1 μm.

To further investigate the possible mechanism of the strain QN1NO-4 extract inhibiting fruit decay of post-harvest strawberry, the spore germination of *C. fragariae* was first analyzed after treatment with extract. The results showed that spore germination was inhibited, and the inhibitory efficiency was gradually increased with the increase of extract concentrations (Figure 5A). In the control group, 95% of spore can germinate, but only 36% and 18% of germination rates were detected in 1 × *EC*_50_ and 2 × *EC*_50_ treatment groups for 12 h, respectively. Especially, more than 4 × *EC*_50_ extract completely inhibited the spore germination of *C. fragariae*. Dense and short hypha at the colony edges were observed in comparison with the sparse and slender mycelia in the control plate (Figure 5B).

We further analyzed the morphological characteristics of *C. fragariae* spores before and after extract treatment. A wizened and distorted surface of spores was observed in the treatment group of 4 × *EC*_50_ extracts for 12 h. The regular and smooth morphology of hypha was displayed in the control group (Figure 5C). By contrast, vacuoles became bigger and disintegrated gradually after treatment with strain H4 extract. The organelle integrity was also broken, and cells showed a cytoplasmic heterogeneity (Figure 5D). In the control group, some complete organelles such as vacuoles and mitochondria could be clearly detected by TEM (Figure 5D).

### Hemolytic Activity Assay of Extract on Eukaryotic Cells

To investigate the toxicity of strain QN1NO-4 extract on eukaryotic cells, the hemolytic activity of extract (1 × *EC*_50_, 2 × *EC*_50_, 4 × *EC*_50_, or 8 × *EC*_50_) on human red blood cells was assayed using the release of hemoglobin after treatment at 37°C for 1 h (Supplementary Figure 1). A percentage of 0.1% of Triton X-100 exhibiting 100% of hemolytic activity was selected as a positive control. No obvious hemolytic activity appeared in all treatment groups. Therefore, extract had non-specific cell lytic activity and toxicity to eukaryotic cells.

### Assay of a Broad-Spectrum Antifungal Activity of Strain QN1NO-4 Extract

**FIGURE 6:**
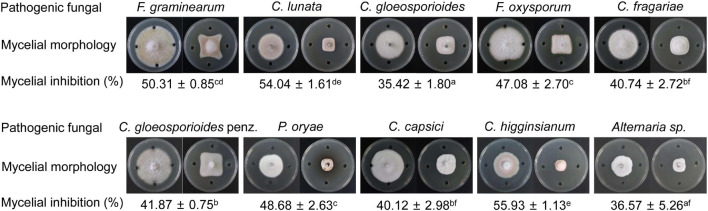
A broad-spectrum antifungal activity of strain QN1NO-4 extract against the selected phytopathogenic fungi.

Based on the prediction of secondary metabolites by genomic sequencing, we further analyzed whether strain QN1NO-4 extract had a broad-spectrum antifungal activity against 10 phytopathogenic fungi selected in our study. After co-incubating for 7 days, the extract exhibited an excellent inhibitory activity for mycelial growth of the selected fungi, ranging from 35 to 56% (Figure 6). The best inhibition activity with 56% was found against *Alternaria* sp., suggesting that the extract had the strongest antifungal activity against the fungi, followed by *C. lunata* (54%) and *F. graminearum* (50%). The minimal inhibition activity of the extract was 35% against *C. gloeosporioides*, indicating that the fungus had the highest tolerable ability for extract treatment.

### Genome Annotation and Metabolite Prediction of Strain QN1NO-4

Functional analysis showed that 2,554, 2,967, and 2,190 out of the identified 3,917 protein-coding genes were annotated into GO, COG, and KEGG categories, respectively (Supplementary Figure 2). For GO categories, the predicted genes were divided into three classes including biological process (27%), cellular components (27%), and molecular function (45%) (Supplementary Figure 2A). For COG categories, these genes were classified into 20 types of four categories. The largest category was metabolism (41%), followed by cellular processes and signaling (18%) as well as information storage and processing (16%). Twenty-five percent of the proportion was classified into the poorly characterized category (Supplementary Figure 2B). KEGG analysis showed that 2,190 of identified protein-coding genes were annotated into 41 pathways (Supplementary Figure 2C). Notably, some pathways including signal transduction, biosynthesis of other secondary metabolites, and metabolism of terpenoids and polyketides were pivotal for disease resistance.

By alignment of antiSMASH software and GenBank, 14 biosynthetic gene clusters containing 457 genes were predicted in the strain QN1NO-4 genome, including three NRPS gene clusters (54 genes), one PKS-like gene cluster (48 genes), one transAT-PKS gene cluster (48 genes), two transAT-PKS-like gene clusters (36 genes), two terpene gene clusters (48 genes), one LAP gene cluster (20 genes), one PKS III gene cluster (48 genes), one beta-lactone gene cluster (50 genes), and one MerR family transcriptional gene cluster (58 genes) (Table 3 and Supplementary Table 6). Among them, eight chemicals produced by gene clusters were involved in the biosynthesis of antimicrobial metabolites, such as bacillibactin, bacilysin, bacillaene, macrolactin, butirosin, difficidin, plantazolicin, and kalimantacin A (Supplementary Figure 3). Four gene clusters (cluster1, cluster2, cluster4, and cluster5) presented a 100% similarity with the known compounds. However, 2 out of 14 clusters exhibited a 13% similarity with the predicted compounds, suggesting that these clusters might be involved in the biosynthesis of novel metabolites.

**TABLE 3:** Cluster number and gene number shown in different cluster types.

Cluster type	Cluster number	Gene number
NRPS	3	54
PKS-like	1	48
TransAT-PKS	1	48
TransAT-PKS-like	2	36
Terpene	2	48
LAP	1	20
PKS III	1	48
Beta-lactone	1	50
MerR family transcriptional	1	58
other	1	47
**Total**	**14**	**457**

*The bold values represent the total of cluster and gene numbers.*

### Bioactive Compound Identification of the Strain QN1NO-4 Extract by GC-MS

**FIGURE 7:**
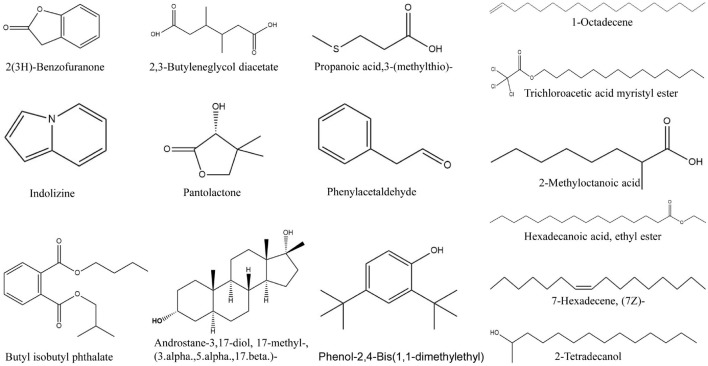
Chemical structures of the identified compounds of the strain QN1NO-4 extract by GC-MS.

GC-MS was employed to analyze the bioactive compounds of the strain QN1NO-4 extract. A total ion current chromatogram is shown in Supplementary Figure 4. Based on retention time, molecular mass, and molecular formula, 15 chemical compounds were identified by the alignment of their mass spectra against the NIST library (Supplementary Table 7). These compounds mainly contained hydrocarbons, acids, pyrrolizidine, esters, and phenol. The predicted chemical structures are shown in Figure 7, including 2-methyloctanoic acid (1), 2,3-butylene glycol diacetate (2), phenylacetaldehyde (3), 7-hexadecene (4), pantolactone (5), propanoic acid, 3-(methylthio) (6), 2(3H)-benzofuranone (7), 1-octadecene (8), 2,4-bis(1,1-dimethylethyl)-phenol (9), indolizine (10), ethyl palmitate (11), 2-tetradecanol (12), trichloroacetic acid myristyl ester (13), 17b-methyl-5a-androstan-3a,17b-diol (14), and butyl isobutyl phthalate (15). The peak areas of compounds represented the portions of different compounds in the strain QN1NO-4 extract. Among these compounds, the peak area of 17b-methyl-5a-androstan-3a,17b-diol was 42%, suggesting that it was a dominant compound in strain QN1NO-4 extracts.

## Discussion

Anthracnose caused by *C. fragariae* is a common and destructive disease of strawberry (Mehmood et al., 2021). Biocontrol has received an increasing attention in controlling post-harvest fruit diseases (Li X.J. et al., 2020). Several endophytes play a vital role in plant protection and plant growth promotion. Many promising endophytic bacteria have been reported as biocontrol candidates against plant pathogens (Morales-Cedeño et al., 2021). Especially, *Bacillus* strains draw more and more attention due to their ability to produce resistant endospores and bioactive metabolites (Zhou et al., 2021). In our present study, a strain labeled QN1NO-4 was isolated from noni fruit and exhibited a strong antifungal ability against *C. fragariae* (Figure 1). According to morphological characteristics as well as genome alignment, strain QN1NO-4 was identified as a novel species of the genus *Bacillus* (Table 1, Figure 2 and Supplementary Tables 3, 4). As potential biocontrol agents, *Bacillus* species had also been successfully used to control other post-harvest diseases of different fruit, such as *Bacillus* species against *B. cinerea* and green mold in strawberry fruit and citrus (Tian et al., 2020; Wang et al., 2021), *B. amyloliquefaciens* against *Penicillium* in oranges and apples (Calvo et al., 2017; Wang et al., 2020; Ye et al., 2021), and *B. atrophaeus* against anthracnose on soursop and avocado (Guardado-Valdivia et al., 2018).

To further elucidate the antifungal mechanism of the strain QN1NO-4 extract against *C. fragariae*, we examined the spore germination, morphology, and ultrastructure of pathogenic cells after extract treatment. Consistent with antifungal activity *in vivo*, more than 4 × *EC*_50_ of strain QN1NO-4 extract completely inhibited the spore germination of *C. fragariae*. In addition, the extract also caused the irregular morphology, cell wall thickening, extensive vacuolization, and intracellular organelle degradation of *C. fragariae* (Figure 5D). Inhibition of spore germination was essential for protecting post-harvest fruit from pathogenic infection (Miyara et al., 2010). A similar germination inhibition of spores was detected in *P. digitatum* treated with *C. lusitaniae* and *P. fermentans*, *P. horianna* treated with *Bacillus* species, and *F. incarnatum* treated with *B. amyloliquefaciens* (Dheepa et al., 2016; Perez et al., 2019; Li Y.G. et al., 2020). Moreover, the toxicity and broad antimicrobial activity of the strain QN1NO-4 extract were also determined (Supplementary Figure 1 and Figure 6), suggesting that it is a promising alternative biological fungicide to control post-harvest diseases.

Natural antimicrobials were an attractive source of controlling post-harvest diseases, mitigating the reliance on synthetic fungicides (Kim et al., 2020). We found that the strain QN1NO-4 extract effectively inhibited mycelial growth of *C. fragariae in vitro*, and the mycelial inhibition rate depended on a dose-dependent manner (Figure 3A). The previous study showed that a novel peptide produced by *B. amyloliquefaciens* W10 had a strong antifungal activity (Zhang et al., 2017). It suggested that high antagonistic components were different from diverse *Bacillus* species. Fox example, extracts from different *Bacillus* species significantly inhibited different mycelial growths of *Fusarium solani*, *P. digitatum*, and *Fusarium incarnatum* (Ahmad et al., 2012; Tian et al., 2020). Moreover, the extract effectively kept the fruit quality of post-harvest strawberry by inhibiting fruit weight loss and TSS decrease (Figures 4C,D). Similarly, weight loss in *L. plantarum*-treated litchis was lower than the control fruit (Martínez-Castellanos et al., 2011).

GC-MS was used to further identify antifungal metabolites of strain QN1NO-4. A total of 15 chemical compounds including hydrocarbons, acids, pyrrolizidine, esters, and phenol were identified (Supplementary Table 7 and Figure 7). Among them, 2(3H)-benzofuranone, 2,4-bis(1,1-dimethylethyl)-phenol, and butyl isobutyl phthalate exhibited high antifungal and antibacterial activities. 2(3H)-benzofuranone produced by the indigenous fungus remarkably inhibited the mycelial growth of *M. oryzae* (Fan et al., 2019). 2,4-bis(1,1-Dimethylethyl)-phenol effectively inhibited the spore germination of *P. cinnamomi* and *Aspergillus* and controlled tomato fungal diseases such as early blight and gray mold (Rangel-Sánchez et al., 2014; Gao et al., 2017). Butyl isobutyl phthalate exhibited an antibacterial activity with an MIC value of 128 g l^–1^ (Hussain et al., 2017). Interestingly, the compounds in the strain QN1NO-4 extract identified by GC-MS were different from those involved in the synthesis of the above gene clusters (Supplementary Tables 6, 7). It might be because the identification method is not enough to identify all metabolites of strain QN1NO-4 (Wei et al., 2020). Thus, further study was necessary to identify specific active metabolites of strain QN1NO-4, contributing to the broad-spectrum antifungal activity.

Considering the deficiency of the GC-MS method, whole-genome analysis was used to predict rare and novel secondary metabolites, which cannot be detected directly in the fermentation broth using the current technology. In our study, the genome of strain QN1NO-4 was sequenced, revealing numerous unknown and known gene clusters of secondary metabolites (Table 3 and Supplementary Figure 3). These gene clusters might be responsible for the production of microbial natural products. For example, the biosynthetic gene clusters encoding for polyketides (PKS) and non-ribosomal peptides (NRPS) were identified. They belonged to a structurally varied group of compounds and participated in the biosynthesis of antifungal compounds (Nimaichand et al., 2015; Prasad et al., 2015). In addition, some antimicrobials were also found such as bacillibactin, bacilysin, fengycin, bacillaene, macrolactin, butirosin, difficidin, plantazolicin, and kalimantacin A (Supplementary Table 6 and Supplementary Figure 3). Bacillibactin was a specific transport system enabling *Bacillus* cells to accumulate and take up limited iron ions from their natural environment (Chen et al., 2009). Bacilysin participated in the antagonistic activity of *B. velezensis* against Gram-negative foodborne pathogens (Nannan et al., 2021). As a potent inhibitor of filamentous fungi, fengycin owned a strong antifungal ability against *V. dahliae* and *B. cinerea* (Li Q.R. et al., 2020; Su et al., 2020). Bacillaene can inhibit prokaryotic protein synthesis (Li Q.R. et al., 2020). Macrolactin effectively inhibited the replication of mammalian Herpes simplex virus and HIV in lymphoblast cells (Gustafson et al., 1989). Butirosin was an aminoglycoside antibiotic produced by *Bacillus circulans* (Llewellyn et al., 2007). Difficidin exhibited a broad-spectrum antibacterial activity (Anthony et al., 2009). Plantazolicin belonged to a type of polyheterocyclic natural product with highly selective and potent activity against anthrax-causing bacteria (Hao et al., 2015). Thistlethwaite et al. (2017) found that kalimantacin showed a selective and high activity against staphylococci with an MIC value of 0.064 g l^–1^. Considering the novel species of strain QN1NO-4 in our study, some unknown function gene clusters might encode some new secondary metabolites. It will be further proved that strain QN1NO-4 has a broad-spectrum resistance to phytopathogen fungi.

## Conclusion

In the present study, a newly isolated strain QN1NO-4 from noni fruit was identified as *B. safensis* sp. and exhibited a strong antifungal ability against *C. fragariae*. Its extract can effectively reduce the fungal disease index and preserve the post-harvest quality of strawberry fruit. Moreover, strain QN1NO-4 extract can significantly inhibit mycelial growth and spore germination of *C. fragariae*, causing morphological and ultrastructure changes of fungal pathogen. Fifteen chemical compounds were identified from strain QN1NO-4 extracts by GC-MS. In addition, the whole-genome analysis revealed that some key function gene clusters in the strain QN1NO-4 genome were involved in the biosynthesis of active secondary metabolites. These diverse gene clusters and metabolites might contribute to its broad-spectrum antifungal activity. Therefore, strain QN1NO-4 is an effective bio-agent for controlling strawberry post-harvest diseases caused by *C. fragariae*.

## Data Availability Statement

The datasets presented in this study can be found in online repositories. The names of the repository/repositories and accession number(s) can be found in the article/Supplementary Material.

## Author Contributions

XL, LZ, JX, and WW developed the ideas and designed the experimental plans. LZ, JX, and WW supervised the research and provided the funding support. XL, MZ, DZ, DQ, CQ, CL, SL, and DX performed the experiments. MZ, DZ, and DQ provided the materials. XL, LZ, and WW analyzed the data and prepared the manuscript. All authors contributed to the article and approved the submitted version.

## Conflict of Interest

The authors declare that the research was conducted in the absence of any commercial or financial relationships that could be construed as a potential conflict of interest.

## Publisher’s Note

All claims expressed in this article are solely those of the authors and do not necessarily represent those of their affiliated organizations, or those of the publisher, the editors and the reviewers. Any product that may be evaluated in this article, or claim that may be made by its manufacturer, is not guaranteed or endorsed by the publisher.

## Footnotes

1
http://www.geneontology.org/

2
http://www.ncbi.nlm.nih.gov/COG

3
http://www.genome.jp/kegg/

4
https://www.ezbiocloud.net/search?Tn=Nocardioides

5
https://www.ezbiocloud.net/tools/ani

## Supplementary Material

Supplementary Figure 1 Hemolytic activity of red blood cells treated with strain QN1NO-4 extract.

Supplementary Figure 2 Genome annotation of strain QN1NO-4.

Supplementary Figure 3 Genome-wide analysis of gene clusters related to the biosynthesis of secondary metabolites in the strain QN1NO-4 genome using the online antiSMASH v4.2.0 software.

Supplementary Figure 4 A total ion current chromatogram of strain QN1NO-4 extract.

Supplementary Table 1 Antifungal activities of 29 isolated bacteria on mycelial growth of *C. fragariae*.

Supplementary Table 2 Antifungal activities of fermentation broth of 10 selected bacteria on mycelial growth of *C. fragariae.*

Supplementary Table 3 Carbon source utilization and nitrogen source utilization of strain QN1NO-4.

Supplementary Table 4 Sensitivity of strain QN1NO-4 to antibiotics.

Supplementary Table 5 Results of average nucleotide identity (ANI).

Supplementary Table 6 Prediction and functional annotation of secondary metabolites of strain QN1NO-4 by alignment with the online antiSMASH v4.2.0 software.

Supplementary Table 7 Compounds identified of extracts from strain QN1NO-4 by GC-MS.
