# Supplementary material for: Biocontrol Ability and Mechanism of a Broad-Spectrum Antifungal Strain Bacillus safensis sp. QN1NO-4 Against Strawberry Anthracnose Caused by Colletotrichum fragariae
Source: Front Microbiol. 2021 Sep 17;12:735732. doi: 10.3389/fmicb.2021.735732 (PMC8486013; doi:10.3389/fmicb.2021.735732)
Supplement: Supplementary Figure 1 — Hemolytic activity of red blood cells treated with strain QN1NO-4 extract. [file Data_Sheet_1.doc]

**Biocontrol ability and mechanism of** **a broad-spectrum antifungal strain *Bacillus safensis* sp. QN1NO-4 against strawberry anthracnose caused by *Colletotrichum fragariae***

**Xiaojuan Li, Miaoyi Zhang, Dengfeng Qi, Dengbo Zhou, Chunlin Qi, Chunyu Li, Siwen Liu, Dandan Xiang, Lu Zhang, Jianghui Xie, Wei Wang**

**
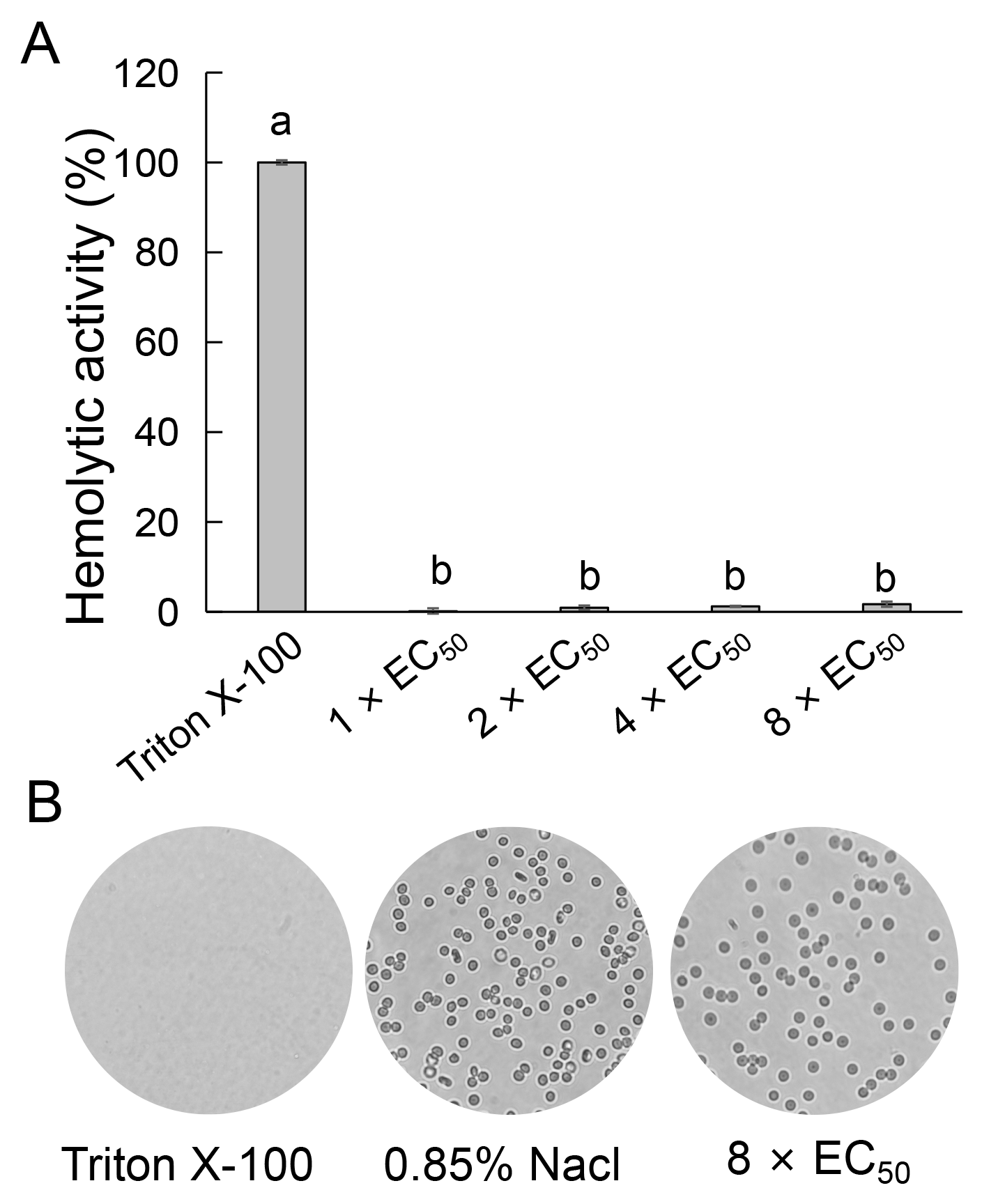
**

**Fig. S1** Hemolytic activity of red blood cells treated with strain QN1NO-4 extract. A, Quantitative analysis of hemolytic activity. Triton X-100 was used as a control. Error bars indicated standard errors of the means from three repeated experiments. Different letters indicated a significant difference according to the Duncan’s multiple range test (P < 0.05). B, Representative picture showing the red blood cells.


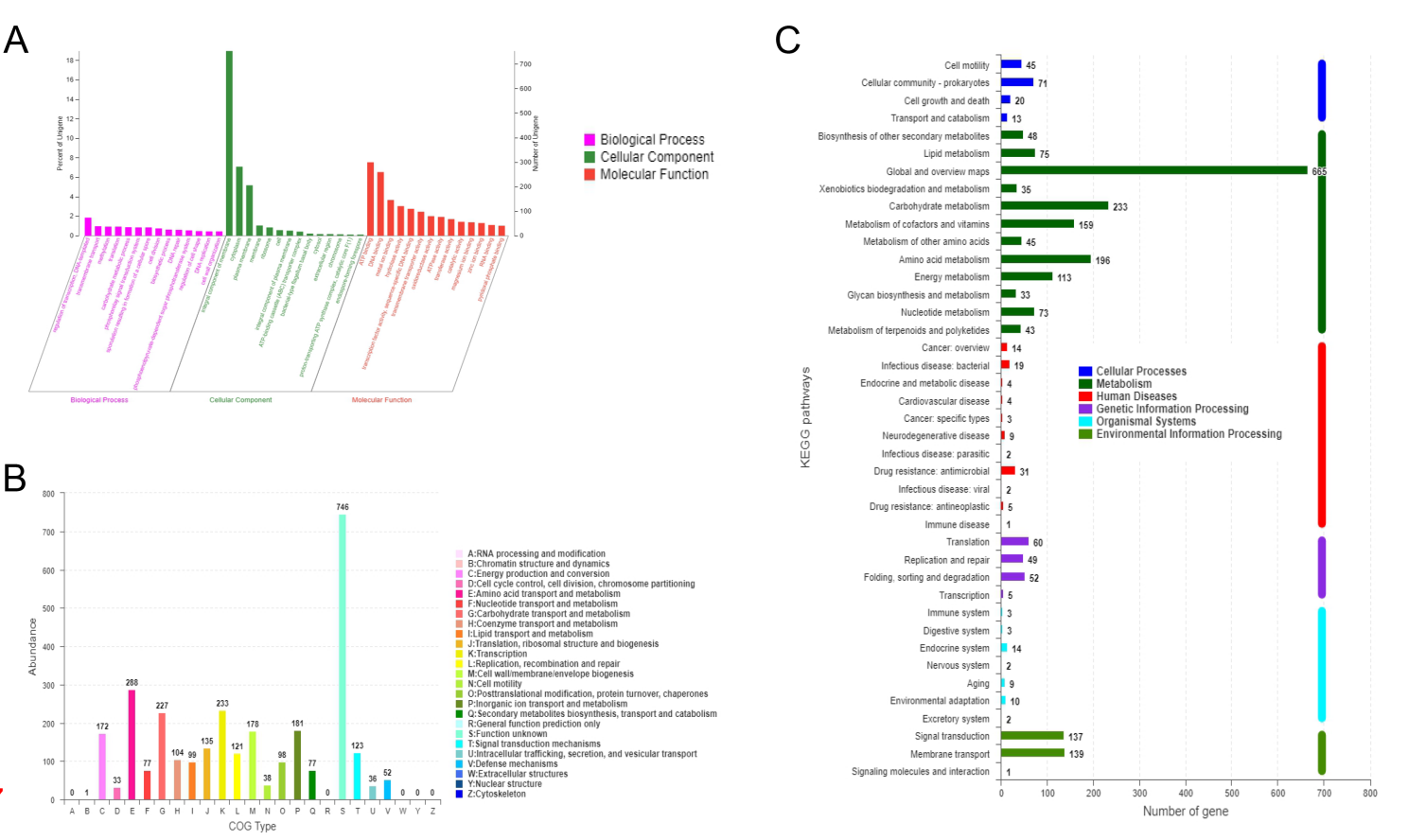


**Fig. S2.** Genome annotation of strain QN1NO-4. A, GO annotation of strain QN1NO-4 genome. B, COG annotation of strain QN1NO-4 genome. C, Pathway annotation of strain QN1NO-4 genome according to the KEGG database.

**
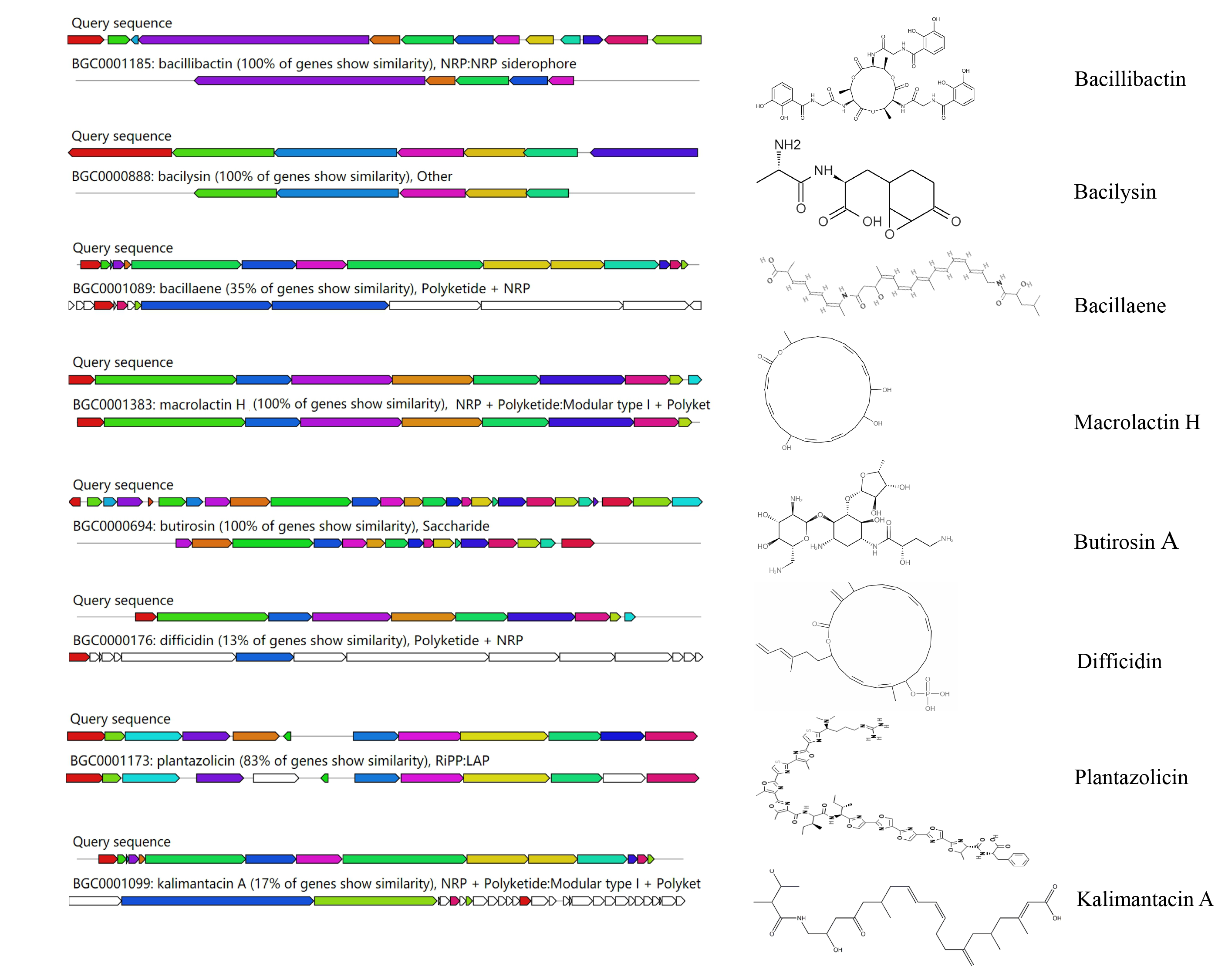
**

**Fig. S3.** Genome-wide analysis of gene clusters related to the biosynthesis of secondary metabolites in the strain QN1NO-4 genome using the online antiSMASH v4.2.0 software.


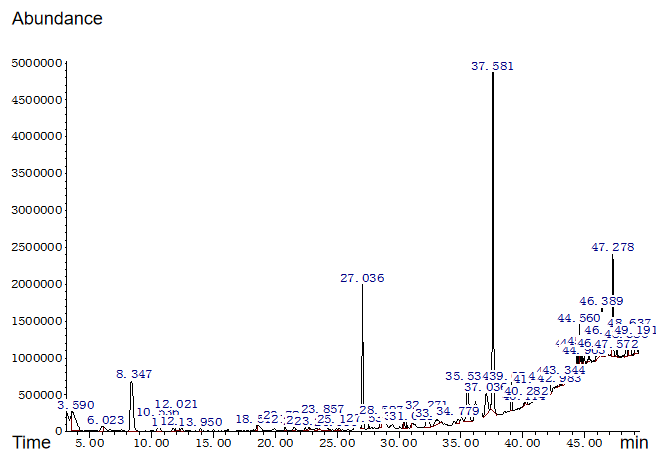


**Fig. S4** A total ion current chromatogram of strain QN1NO-4 extract

| **Table S1** Antifungal activities of 29 isolated bacteria on mycelial growth of *C. fragariae* |
| --- |
| | **Strain NO.** | **Inhibition rate（%）** | **Strain NO.** | **Inhibition rate （%）** | | --- | --- | --- | --- | | QN1NO-1 | 67.30±0.75 | QN1NO-20 | 68.77±1.19 | | QN1NO-2 | 66.24±1.49 | QN1NO-21 | 67.72±1.62 | | QN1NO-4 | 70.17±1.24 | QN1NO-22 | 69.41±0.93 | | QN1NO-5 | 67.72±0.45 | QN1NO-23 | 67.51±1.27 | | QN1NO-6 | 68.77±0.30 | QN1NO-24 | 68.77±1.13 | | QN1NO-8 | 66.24±1.49 | QN1NO-25 | 67.08±0.60 | | QN1NO-9 | 65.82±0.30 | QN1NO-26 | 65.19±3.73 | | QN1NO-10 | 65.19±0.75 | QN1NO-27 | 68.98±1.04 | | QN1NO-11 | 58.86±1.84 | QN1NO-29 | 67.93±1.79 | | QN1NO-12 | 66.66±0.92 | QN1NO-30 | 66.03±0.85 | | QN1NO-14 | 66.03±1.74 | QN1NO-31 | 69.83±1.04 | | QN1NO-15 | 71.94±0.45 | QN1NO-32 | 68.35±1.49 | | QN1NO-16 | 67.51±0.30 | QN1NO-34 | 61.18±0.60 | | QN1NO-17 | 65.82±1.53 | QN1NO-35 | 57.17±1.04 | | QN1NO-19 | 64.76±0.45 |  |  | |

| **Table S2 Antifungal activities of** **fermentation broth of 10 selected bacteria on mycelial growth of *C. fragariae*** |
| --- |
| | **Strain NO.** | **Inhibition rate（%）** | | --- | --- | | QN1NO-1 | 15.25±0.50 | | QN1NO-3 | 33.51±1.25 | | QN1NO-4 | 35.11±0.93 | | QN1NO-18 | 19.33±1.27 | | QN1NO-21 | 24.11±2.01 | | QN1NO-29 | 23.05±0.60 | | QN1NO-31 | 22.34±0.73 | | QN1NO-32 | 24.47±1.50 | | QN1NO-35 | 27.66±2.01 | |

**Table S3** [Carbon](../../../../C:/Users/Administrator/AppData/Local/youdao/dict/Application/8.5.3.0/resultui/html/index.html#/javascript:;) [source](../../../../C:/Users/Administrator/AppData/Local/youdao/dict/Application/8.5.3.0/resultui/html/index.html#/javascript:;) [utilization](../../../../C:/Users/Administrator/AppData/Local/youdao/dict/Application/8.5.3.0/resultui/html/index.html#/javascript:;) and nitrogen [source](../../../../C:/Users/Administrator/AppData/Local/youdao/dict/Application/8.5.3.0/resultui/html/index.html#/javascript:;) [utilization](../../../../C:/Users/Administrator/AppData/Local/youdao/dict/Application/8.5.3.0/resultui/html/index.html#/javascript:;) of strain QN1NO-4.

| Characteristics | Result |
| --- | --- |
| [Carbon](../../../../C:/Users/Administrator/AppData/Local/youdao/dict/Application/8.5.3.0/resultui/html/index.html#/javascript:;) [source](../../../../C:/Users/Administrator/AppData/Local/youdao/dict/Application/8.5.3.0/resultui/html/index.html#/javascript:;) [utilization](../../../../C:/Users/Administrator/AppData/Local/youdao/dict/Application/8.5.3.0/resultui/html/index.html#/javascript:;) |  |
| D- Galactose | +++ |
| D- Mannose | ++ |
| D-Fruotose | ++ |
| D-Glucose | +++ |
| D-Mannitol | +++ |
| D-Melezitose | ++ |
| D-Ribose | + |
| L-Arabinose | ++ |
| L-Rhamnose | ++ |
| Maltose | +++ |
| Myo-Inositol | +++ |
| Raffinose | +++ |
| Saccharose | +++ |
| [Soluble](../../../../C:/Users/Administrator/AppData/Local/youdao/dict/Application/8.5.3.0/resultui/html/index.html#/javascript:;) [starch](../../../../C:/Users/Administrator/AppData/Local/youdao/dict/Application/8.5.3.0/resultui/html/index.html#/javascript:;) | ++ |
| Sorbitol | +++ |
| D-Xylopyranose | ++ |
| α- Lactose | +++ |
| D-Cellose | + |
| Xylan | ++ |
| Trehalose | +++ |
| Nitrogen [source](../../../../C:/Users/Administrator/AppData/Local/youdao/dict/Application/8.5.3.0/resultui/html/index.html#/javascript:;) [utilization](../../../../C:/Users/Administrator/AppData/Local/youdao/dict/Application/8.5.3.0/resultui/html/index.html#/javascript:;) |  |
| Ammonium Acetate | + |
| [Ammonium](../../../../C:/Users/Administrator/AppData/Local/youdao/dict/Application/8.5.3.0/resultui/html/index.html#/javascript:;) [Sulfate](../../../../C:/Users/Administrator/AppData/Local/youdao/dict/Application/8.5.3.0/resultui/html/index.html#/javascript:;) | ++ |
| Creatine | ++ |
| Glycine | + |
| L-Arginine | ++ |
| L-Asparagine | ++ |
| L-Cysteine | + |
| L-Glutamate | - |
| L-Histidine | ++ |
| L-Methionine | + |
| L-Phenylalanine | ++ |
| L-Tryptophan | + |
| L-Tyrosine | - |
| L-Valine | +++ |

+++, good growth; ++, moderate growth; +, poor growth; -, no growth.

**Table S4** Sensitivity of strain QN1NO-4 to antibiotics.

| Characteristics | Result |
| --- | --- |
| Amikacin | S |
| Ampicillin | S |
| Carbenicillin | S |
| Cefamezin | S |
| Cefoperazone | S |
| Ceftazidime | S |
| Ceftriaxone | S |
| Cefuroxim | S |
| Cephalexin | S |
| Cephradine | S |
| Doxycycline | S |
| Erythromycin | S |
| Gentamicin | S |
| Kanamycin | S |
| Minocycline | S |
| Neomycin | S |
| Oxacillin | S |
| Penicillin | R |
| Piperacillin | R |
| Tetracycline | S |

S, sensitivity; R, resistance.

**Table S5** Results of average nucleotide identity (ANI)

| Metric | *Bacillus siamensis*  KCTC 13613 | *Bacillus subtilis*  NCIB 3610 |
| --- | --- | --- |
| ANI value (%) | 94.38 | 77.07 |
| Genome A length (bp) | 3,887,220 | 3,887,220 |
| Genome B length (bp) | 3,757,680 | 4,298,280 |
| Average aligned length (bp) | 2,684,897 | 1,898,402 |
| Genome A coverage (%) | 69.07 | 48.84 |
| Genome B coverage (%) | 71.45 | 44.17 |

**Table S6** Prediction and functional annotation of [secondary](javascript:;) [metabolite](javascript:;)s of strain QN1NO-4 by alignment with the online antiSMASH v4.2.0 software

| Number | Type | Location (bp) | Predicted compounds | Similarity | Functional annotation | References |
| --- | --- | --- | --- | --- | --- | --- |
| Cluster1 | NRPS | 73348-125142 | Bacillibactin | 100% | Antimicrobial and antibacterial activities | Chen et al., 2009 |
| Cluster2 | Other | 649270-690689 | Bacilysin | 100% | Antibacterial activity | Vairagkar et al., 2021 |
| Cluster3 | Betalactone | 0-87192 | Fengycin | 73% | Antifungal and antibacterial activities | Su et al., 2020 |
| Cluster4 | MerR family Transcriptional | 150766-260377 | Bacillaene | 100% | Antibacterial activity | Li et al., 2020c |
| Cluster5 | TransAT-PKS | 479641-567872 | Macrolactin | 100% | Antiviral and cytotoxic activities | Gustafson et al., 1989 |
| Cluster6 | Terpene | 124387-145128 | - | - |  |  |
| Cluster7 | PKS-like | 227514-268759 | Butirosin A,B | 7% | Antibiotic activity | Nicholas et al., 2007 |
| Cluster8 | TransAT-PKS-like | 0-45777 | Difficidin | 53% | Antimicrobial and antibacterial activities | Anthony et al., 2009 |
| Cluster9 | T3PKS | 161344-202445 | - | - |  |  |
| Cluster10 | LAP | 23790-45973 | Plantazolicin | 91% | Antibiotic activity and cytotoxicity | Hao et al., 2015 |
| Cluster11 | Terpene | 50633-72517 | - | - |  |  |
| Cluster12 | TransAT-PKS-like | 0-23533 | Kalimantacin A | 6% | Antibiotic activity | Thistlethwaite et al., 2017 |
| Cluster13 | NRPS | 0-13132 | Fengycin | 13% | Antimicrobial activity | Su et al., 2020 |
| Cluster14 | NRPS | 0-10293 | - | - |  |  |

**Table S7** Compounds identified of extracts from strain QN1NO-4 by GC-MS.

| Compound’s name | RT (min) | MM | Area (%) | MF | Probability (%) | Activity | References |
| --- | --- | --- | --- | --- | --- | --- | --- |
| 2-Methyloctanoic acid | 8.347 | 158.131 | 15.517 | C_9_H_18_O_2_ | 64 | No activity reported |  |
| 2,3-Butyleneglycol diacetate | 12.44 | 174.089 | 0.629 | C_8_H_14_O_4_ | 72 | No activity reported |  |
| Phenylacetaldehyde | 13.95 | 120.058 | 0.402 | C_8_H_8_O | 90 | Antiproliferation | Choi et al., 2020 |
| 7-Hexadecene | 18.514 | 224.25 | 0.542 | C_16_H_32_ | 97 | Aggregation-Sex Pheromone | Silva et al., 2018 |
| Pantolactone | 20.787 | 130.063 | 0.894 | C_6_H_10_O_3_ | 83 | Antiplasmodial | Baldé et al., 2020 |
| Propanoic acid,3-(methylthio) | 21.492 | 120.025 | 0.771 | C_4_H_8_O_2_S | 64 | No activity reported |  |
| 2(3H)-Benzofuranone | 23.345 | 134.037 | 0.442 | C_8_H_6_O_2_ | 76 | Antifungal | Fan et al., 2019 |
| 1-Octadecene | 23.857 | 252.282 | 6.597 | C_18_H_36_ | 99 | No activity reported |  |
| 2,4-bis(1,1-dimethylethyl)-phenol | 27.036 | 206.167 | 16.349 | C_14_H_22_O | 97 | Antifungal | Rangel-Sánchez et al., 2014 |
| Indolizine | 27.531 | 117.058 | 0.358 | C_8_H_7_N | 93 | Anticancer | Park et al., 2017 |
| Ethyl palmitate | 32.271 | 284.272 | 4.462 | C_18_H_36_O_2_ | 96 | Antiefflux pump activity | Khosravani et al., 2020 |
| 2-Tetradecanol | 33.06 | 214.23 | 2.058 | C_14_H_30_O | 64 | No activity reported |  |
| Trichloroacetic acid myristyl ester | 37.036 | 358.123 | 5.966 | C_16_H_29_Cl_3_O_2_ | 64 | No activity reported |  |
| 17b-Methyl-5a-androstane-3a,17b-diol | 37.581 | 306.256 | 42.482 | C_20_H_34_O_2_ | 89 | No activity reported |  |
| Butyl isobutyl phthalate | 39.074 | 278.152 | 2.530 | C_16_H_22_O_4_ | 95 | Antibacterial, Antitumor | Hussain et al., 2017 |
